# Language in the brain at rest: new insights from resting state data and graph theoretical analysis

**DOI:** 10.3389/fnhum.2014.00228

**Published:** 2014-04-28

**Authors:** Angela M. Muller, Martin Meyer

**Affiliations:** ^1^Research Unit for Neuroplasticity and Learning in the Healthy Aging Brain, Psychological Institute, University of ZurichZurich, Switzerland; ^2^International Normal Aging and Plasticity Imaging CenterZurich, Switzerland; ^3^URPP Dynamics of Healthy Aging, University of ZurichZurich, Switzerland; ^4^Cognitive Psychology Unit, Department of Psychology, University of KlagenfurtKlagenfurt, Austria

**Keywords:** default mode network, graph theoretical analysis, intrinsic connectivity fMRI, language, networks, resting state, task free paradigm

## Abstract

In humans, the most obvious functional lateralization is the specialization of the left hemisphere for language. Therefore, the involvement of the right hemisphere in language is one of the most remarkable findings during the last two decades of fMRI research. However, the importance of this finding continues to be underestimated. We examined the interaction between the two hemispheres and also the role of the right hemisphere in language. From two seeds representing Broca's area, we conducted a seed correlation analysis (SCA) of resting state fMRI data and could identify a resting state network (RSN) overlapping to significant extent with a language network that was generated by an automated meta-analysis tool. To elucidate the relationship between the clusters of this RSN, we then performed graph theoretical analyses (GTA) using the same resting state dataset. We show that the right hemisphere is clearly involved in language. A modularity analysis revealed that the interaction between the two hemispheres is mediated by three partitions: A bilateral frontal partition consists of nodes representing the classical left sided language regions as well as two right-sided homologs. The second bilateral partition consists of nodes from the right frontal, the left inferior parietal cortex as well as of two nodes within the posterior cerebellum. The third partition is also bilateral and comprises five regions from the posterior midline parts of the brain to the temporal and frontal cortex, two of the nodes are prominent default mode nodes. The involvement of this last partition in a language relevant function is a novel finding.

## Introduction

One of the best established functional lateralization of the human brain is the superiority of the left hemisphere in language. Nevertheless, the term lateralization only implies that the processing of language in the brain is not symmetric, but this does not imply that the right hemisphere contributes nothing to language processing. Indeed, in their two meta-analyses of language related fMRI-studies Vigneau et al. (2006, 2011) found that at least 59 out of the 128 articles also reported right sided activation peaks during sentence or context processing tasks. Even though the relatively strong involvement of the right hemisphere in language is one of the most remarkable and intriguing findings of the last two decades of fMRI research, most researchers investigating the organization of language in the brain still place their focus mostly on the contribution made by the left hemisphere. This is evidenced by a recent and very comprehensive review of the PET and fMRI language studies during the last 20 years (Price, 2012), which only discusses the right cerebellum when referring to language processing in the right side of the brain. Because of this focus on the left hemisphere's role in language processing, the contribution of the right hemisphere as well as the interaction between the two hemispheres to accomplish this uniquely human function is still not very well-understood.

Therefore, we aimed to investigate how the left dominant and the right hemisphere cooperate in order to process language. For that purpose, we departed from the traditional task evoked fMRI paradigm, and employed a resting state or functional intrinsic connectivity analysis instead. The task-evoked fMRI paradigm focuses on the brain regions that show an event-related BOLD activity over a significant threshold. However, such task-induced BOLD signals generally exceed the permanently ongoing baseline activity of the brain by no more than 5%. The remaining 95% of intrinsic BOLD signal modulation is interpreted as neural noise, which is then minimized by averaging the data and subtracting it from the analysis (Fox and Raichle, 2007; Raichle, 2010). In contrast, the functional resting state technique studies explicitly the interdependence of that spontaneous, low frequent BOLD signal modulation between the brain regions. It has thus the potential to detect language relevant regions whose role for language processing may hitherto have been underestimated, because their activity was just not strong enough to surpass the activation threshold of the former traditional task evoked fMRI paradigms.

Resting state patterns seem to be quite similar to the task-induced activation patterns found in the standard task-evoked fMRI paradigm, and are also remarkably reliable across participants and time (Cordes et al., 2000; Damoiseaux et al., 2006; De Luca et al., 2006; Smith et al., 2009). However, the biological relevance of resting state networks (RSNs) is still not fully understood. Wig et al. (2011) interpret these resting state relationships as being mediated by a Hebbian-like mechanism in which the continual recruitment of a set of areas for a common purpose results in changes in the synaptic efficiencies between these regions. A somewhat more dynamic perspective on this phenomenon is taken by Fox and Raichle (2007) and Deco et al. (2011), as they argue that the spontaneous activity of these RSN may represent a dynamic prediction about expected use with correlations occurring between regions that are likely to be used together in the future. Sadaghiani et al. (2010) emphasize the fact that these low frequent fluctuations are not restricted to the brain at rest, but are also present in the actively engaged brain. Stressing especially the itinerant quality of the intrinsic fluctuations of the BOLD signal, Sadaghiani et al. (2010) speculate that this quality may reflect the dynamic nature of the underlying internal model, which may not be locked in a stationary mode, but rather remains malleable by continuously exploring hypotheses pertinent to future experience and action.

In this way, RSNs are the functional base from which the brain generates behavior. Thus, even when there is no doubt that these intrinsic networks of the brain are modulated when the brain is performing an actual task (Arbabshirani et al., 2013; Rehme et al., 2013), we feel confident that analysing RSNs and their relationships is an important complement in order to fully understand how the brain works. This aspect has thus far rather been neglected in the fMRI research on language, hence our investigation placed primary focus on it.

We decided to investigate the dynamic interaction of one of the most prominent regions related to language production as well as perception, the Broca's area in the left inferior frontal gyrus, with the whole brain under task free conditions. Our aim was to identify all regions that might be relevant for spoken language, and to form hypotheses how these regions may organize in sub-networks with distinct functions. For this purpose, we first conducted a seed correlation analysis (SCA) on resting state data, in order to identify all regions that form a RSN with Broca's area. In a SCA the relationship, i.e., the correlation between the time series of a seed/region of interest (ROI) and all other gray matter (GM) voxels, is computed. The result is a connectivity map showing clusters of voxels whose time series are significantly correlated with the seed. In a second step, we transformed the peak voxels of these clusters into network nodes with the aim to investigate the characteristic of this functional sub-network and the roles and relationships of its constituent nodes by means of graph theoretical analyses (GTA).

There exists already a number of studies on language that have used resting state data and SCA to investigate how the brain processes spoken language. The majority of these studies though is based on the assumption that language processing in the brain is already well-understood. Therefore, they were not primarily designed to investigate language processing, but to address methodical problems, e.g., the relationship of functional connectivity to structural connectivity or the linking of intrinsic connectivity to effective functional connectivity (Cordes et al., 2000; Hampson et al., 2002; Morgan et al., 2009; Kelly et al., 2010). So far, only six studies (Lohmann et al., 2010; Xiang et al., 2010; Friederici et al., 2011; Turken and Dronkers, 2011; Tomasi and Volkow, 2012; Antonenko et al., 2013) used resting state analysis to identify and explore the language related networks. Xiang et al. (2010) and Tomasi and Volkow (2012) both followed purely theoretical considerations and defined the seeds based on anatomical landmarks like Wernicke's area or Borca's area (Tomasi and Volkow, 2012) and not on a priori empirical evidence provided by task-evoked fMRI studies. This approach comes with a methodical “caveat,” as one may wonder to what degree anatomical seeds indeed represent a language function. For instance, the notion of Wernicke's area is by all means anatomically ill and inconsistently defined (Bogen and Bogen, 1976). Turken and Dronkers (2011) chose the location and the size of their seeds in accordance with results from a former Voxel-Based-Symptom-Lesion-Mapping (VBSLM) study of lesion-related deficits in aphasics. Accordingly, their seeds comprise large macro-anatomical regions, such as the posterior part of the middle temporal gyrus. It is therefore a reasonable assumption that the averaged BOLD signal from such a large seed rather represents a mixture of many different functions. Thus, RSNs modeled by such seeds might have a restricted explanatory value for language processing. In contrast, Lohmann et al. (2010) and Friederici et al. (2011) both used activation peaks of previous task-evoked fMRI language studies as seeds which are more likely to represent brain regions effectively involved in language processing than anatomical seeds. Another common feature of these two studies is that the authors did not use resting state data. Assuming that there is a linear addition of task-related activity on top of ongoing intrinsic activity (Fox et al., 2006), Lohmann et al. (2010) and Friederici et al. (2011) used existing data from former event-related language studies and analyzed the information conveyed by the low frequency components of the regressing residuals ε of the general linear model (GLM). For methodological reasons this approach is disadvantageous. However, Fair et al. (2007) demonstrated that the results of such low frequency fluctuation paradigms show distinct quantitative differences in comparison to intrinsic connectivity results. There seem to be non-linear task effects that the GLM is not able to eliminate. Therefore, results generated by this approach should be interpreted carefully (Fair et al., 2007).

Based on the observations of Cordes et al. (2000), De Luca et al. (2006), and Smith et al. (2009) that the spatial distribution of RSNs is very similar to the task-induced activation patterns found in the standard task evoked fMRI paradigm, we predicted that the cluster map of the SCA originating from Broca's area will show a language related pattern involving the whole left perisylvian cortex (i.e., all areas encompassing the anterior and posterior parts of the Sylvian Fissure, namely the left superior temporal gyrus, the left supramarginal gyrus, and in the frontal lobe the left inferior frontal gyrus covering the pars opercularis, and the pars triangularis) and additionally the extra-perisylvian regions of the left angular gyrus and the left middle temporal gyrus. Based on evidence from previous event-related fMRI studies on language which suggest that the right side of the human brain is also essential for the processing of language, we predicted to find clusters in regions of the right perisylvian region (Meyer et al., 2000; Van Ettinger-Veenstra et al., 2010; Vigneau et al., 2011; Van Ettinger-Veenstra et al., 2012). SCA with resting state data has a serious limitation, in that the resulting cluster-maps only illustrate the relationship between the seeds and the clusters, but not the relationships between clusters. We therefore performed various GTAs in order to be able to investigate the dependencies and relationships of the regions from the SCA cluster map. The main GTA parameters allowed us to describe the influence, the capacity for segregated and/or integrated information processing of every single node within that network as also the degree of modularity of the overall network.

## Materials and methods

### Participants

Twenty-four healthy male adults with no psychiatric or neurologic history participated in this study. All of the participants were right-handed (controlled with the handedness-questionnaire of Annett, 1970), Swiss-German or German native speakers, and none of them was bilingual. All participants gave informed consent to the experiment under the approval of the local ethics committee and were paid for their participation. Because of significant motion artifacts in the data sets only the data of 18 subjects (age 21–49 years; ∅ = 31.7; *SD* = 8.36) were used for the analyses of this study.

### MRI-acquisition

All MRI-Data were acquired on a Philipps 3T Achieva Medical Scanner with a Philipps 32 channel head-coil at the Psychiatrisches Universitätsspital Zürich (PUK).

Task-free blood oxygenation level-dependent signals (BOLD) were recorded for each participant for 10 min. For this part of the data acquisition, the participants were instructed to relax but also to lay as motionless as possible, to keep the eyes open and to think on nothing in particular. Instantly after the 10 min scan, each participant was asked if he had fallen asleep. All participants affirmed that they were fully awake during the 10 min of the task free scan.

The acquisition parameters for the single shot EPI sequence were: 200 volumes per participant covering the whole brain with a *TR* = 3000 ms, *TE* = 35 ms, Flip Angle = 77°; 48 transverse slices (FOV 240 × 144 × 240 mm^3^, matrix size 80 × 80, interleaved acquisition without gap, isotropic voxel size 3 mm, aligned to the AC-PC-line). The first four volumes were discarded allowing for T1 saturation effects, leaving 196 volumes for the analysis of the endogenous low frequency BOLD signals dynamics during a no task condition.

Directly after the functional time series a T1 weighted FSE-SENSE sequence was acquired for each participant with the following parameters: *TR* = 8 ms, *TE* = 3.7 ms, Flip Angle = 8° (FOV 240 × 160 × 240 mm^3^, matrix size 256 × 256, voxel size 0.94 × 0.94 × 1 mm) 160 slices per volume.

### Preprocessing

All preprocessing steps were performed with the SPM8 software (http://www.fil.ion.ucl.ac.uk/spm) implemented in Matlab (MATLAB R 2011B, The MathWorks Inc.). First, the fMRI time series were slice time corrected for the interleaved acquisition and then, using an affine transform implemented in SPM, realigned to the first functional scan to correct for potential head movements during the task free scan. SCA on voxel level as well as GTA are both very sensible to motion caused artifacts (Power et al., 2012). Therefore, six datasets with motion artifacts exceeding 1 mm translation or 1° rotation, were excluded from the further analysis. The next steps were: (1) Coregistration of the T1 weighted anatomical image to the mean of the EPI-data for each participant to ensure maximal spatial overlap. (2) Segmentation of the T1 weighted anatomical image into GM, white matter (WM), and cerebrospinal (CSF) tissue in native space. (3) Generating a study-population specific GM template using DARTEL implemented in SPM 8 (Ashburner, 2007), that allows for a high dimensional and non-linear registration of the anatomical and functional images and their subsequent normalization to the MNI-template. The functional and the anatomical data were resampled to a 2 mm isotropic voxel-size during this step. For the GTA the EPI were analyzed unsmoothed. (4) Smoothing of the EPI data with an isotropic Gaussian kernel (FWHM 4 mm) for the SCA.

### Seed correlation analysis

All the resting state analyses were performed with the Conn Toolbox (version 13 l) (http://web.mit.edu/swg/software/ or http://www.nitrc.org/projects/conn) for SPM (Whitfield-Gabrieli and Nieto-Castanon, 2012). Since the spontaneous, coherent, and low frequent fluctuations of the BOLD-signal are used for the resting state analyses, the BOLD time series for each participant were extracted and band-pass-filtered (0.008–0.08 Hz). As non-neural low frequent (<0.1 Hz) signals such as heart rate or respiration are able to modulate the BOLD-signal, they may likewise influence the resting state maps. Hence, a further important step is to correct for these physiological confounding signals. The Conn Toolbox accomplishes this by means of the CompCor method (Behzadi et al., 2007).

Next, correlation maps on voxel level were constructed by correlating the averaged BOLD-signal dynamic of the previously defined two regions of interest (cf. next paragraph) with the BOLD-signal of every other single voxel over the 10 min duration of the scan for each participant. To enforce a Gaussian distribution of the correlation data the Pearson's correlations *r* were then transformed to z-scores using the Fisher *r* to *z* transformation. For the group level analysis the toolbox Conn 13 l implements contrasts for analyses at the voxel level as repeated-measures analyses by using ReML estimation of covariance components that are evaluated through F-statistical parameter maps. An extension threshold for the clusters: *p* < 0.01 FDR corrected and a height threshold for the peak voxel: *p* < 0.01 FWE corrected was used for the computing of the resting state cluster map for the two seeds in Broca's area (Chumbley et al., 2010).

#### Definition of the seeds for the SCA

Seeds that are based on a priori evidence from task evoked fMRI experiments generate functionally more plausible resting state cluster maps for complex cognitive functions than seeds defined by macro-anatomical boundaries (Smith et al., 2011). Since Lohmann et al. (2010) and Friederici et al. (2011) had investigated a question similar to ours and had used seeds from previous task evoked language comprehension paradigms, we decided to pick one seed from each study. We chose the seed in the left pars triangularis (−53 +20 +15) from Friederici et al. (2011) and the seed in the left pars opercularis (−46 +16 +8) from Lohmann et al. (2010). These two seeds together represent Broca's area, one of the most prominent brain regions associated with the perception and production of spoken language (Vigneau et al., 2006; Friederici, 2011, 2012; Price, 2012). Furthermore, the coordinates of the two seeds had shown a significant activation during a syntactic task in a previous fMRI study of Friederici et al. (2006) and thus seem to be representative for the same aspect of language, i.e., for recursive structure.

To reconstruct the two seeds, the normalized GM images of all 18 subjects were averaged and then thresholded at 0.01 to generate a binary mask on which two spherical ROIs (4 mm radius) were drawn using the drawing tool of MRIcron (http://www.mccauslandcenter.sc.edu/mricro/mricron/).

Because the coordinates of both seeds were originally given in the Talairach space, the “tal2mni” Routine of Matthew Brett (http://imaging.mrc-cbu.cam.ac.uk/imaging/MniTalairach) was used for their transformation into MNI-Space.

### Corroboration of the resting state network for broca's area by an independent component analysis and the neurosynth database

To some degree the choice of a seed for a SCA is always an arbitrary decision. To confirm the neurobiological reliability of the SCA cluster map by a purely data driven approach, we therefore performed an independent component analysis (ICA) on group level using the MATLAB based toolbox GIFT (http://icatb.sourceforge.net/).

ICA is a statistical method of blind signal source separation. Assuming a generative model and a linear mixture of independent sources, it works with higher order statistics in order to maximize the spatial or temporal independence of the data and to identify the independent components hidden in the signal (Calhoun et al., 2001). In contrast to a SCA an ICA does not need an a priori assumption about the regions subserving a particular brain function or about the estimated time course of the function of interest. Therefore, it may reveal regions involved in the function under investigation that would not have been detected otherwise. In a first step, the preprocessed time series of all participants were concatenated (Calhoun et al., 2001). Secondly this aggregated data set was reduced by means of a PCA and then an ICA estimation was performed in order to decompose the reduced data into these 70 components using the Infomax algorithm (Calhoun et al., 2001). Thirdly, the corresponding components for each subject were calculated by back reconstruction (Calhoun et al., 2001). In a fourth next step, the independent components on group level were sorted by means of spatial correlation analysis. As template for this spatial sorting served the cluster mask that was generated by the previous SCA, and the component with the highest correlation coefficient was identified. The significance of that correlation was corrected for multiple comparisons by means of the same procedure proposed by Smith et al. (2009).

The relevance of the observed SCA cluster map for language was evaluated with the NeuroSynth database (http://www.neurosynth.org). NeuroSynth is an internet based platform for large-scale, automated synthesis of functional magnetic resonance imaging (fMRI) data extracted from over 4393 published articles, which cover over 147,493 activations (Yarkoni et al., 2011). The most appealing advantage of that platform is its ability to quantitatively distinguish forward inference from reverse inference. While a forward inference is able to identify brain regions that are consistently activated during a cognitive function in question, the reverse inference identifies brain regions that are preferentially involved in that specific function. A forward inference may not be very specific because some brain regions like the anterior cingulate are consistently active during many different cognitive tasks. However, by means of reverse interference it is possible to quantitatively identify cognitive states or functions from observed patterns of brain activity and thus to make quite specific statements about the role of a region in the function under question. Also the NeuroSynth framework allows one to compute the posterior probability, i.e., to quantify the probability, that an activated voxel is associated with a particular cognitive function (Yarkoni et al., 2011). We used the NeuroSynth framework to compute the reverse inference *z*-value of the two seeds used for the SCA for the NeuroSynth feature “language” as well to test their posterior probability for that feature. As database for the NeuroSynth feature “language” serves an automated meta-analysis of the 553 task evoked fMRI studies. Also the NeuroSynth framework provides an option to generate and download a z mask for the investigated feature thresholded at *p* = 0.05 (FWE corrected). We used that mask “language” from NeuroSynth as a template to perform a second spatial sorting of the 70 independent components from the ICA and then compared these results with the results of the previous sorting by means of the cluster mask, that was generated by the SCA.

### Definition of the nodes, construction of an adjacency matrix, and modeling a graph-network

The cluster map of the two seeds in Broca's area showed totally 24 clusters (*k* = 56–6454 voxels). The peak voxels of all 24 clusters were used as centroids to build 24 spherical nodes (4 mm radius). The node corresponding to the peak voxel of the spatially most extensive cluster (6454 voxels) which covered the surface from the left prefrontal cortex into the middle temporal gyrus overlapped with the former original seed in the pars opercularis. Therefore, it was replaced by the two original seeds from the precedent SCA. Thus, the finally analyzed graph network of the resting state of the Broca region consisted of 25 nodes in total.

The next step was the creation of a positively and negatively weighted adjacency matrix of the 25 nodes. With the exception of the first modularity analysis, for which we used an unthresholded weighted adjacency matrix, all further GTA were performed with weighted adjacency matrices that were thresholded over a range from *r* = 0.20 to 0.27 (Fisher transformed) in steps of 0.01.

The following criteria were used to define the range for the investigated thresholds:

- The network of our empirical data should be fully connected, i.e., every node should be integrated in the network by at least one edge. That criterion was met at a threshold of *r* = 0.27.
- The network should meet the criteria for a small world network, if possible. A network possesses small world characteristics when its global efficiency approximates the global efficiency of an equivalent random network and when its local efficiency clearly surpasses that of an equivalent random network (Latora and Marchiori, 2001, 2003; Bullmore and Sporns, 2009, 2012; Rubinov and Sporns, 2010).
- The network of our empirical data should be as cost efficient as possible. The costs of a graph network are defined as the ratio of all existing edges in the actual network and all possible edges between the nodes of the network. To compute the cost efficiency or the wiring costs of a network, one subtracts the cost of a network from the global efficiency of the network. Additionally, the wiring costs of an efficient network should not exceed the value of 0.5. If the wiring costs were above this limit, then the properties of the small-world network would merge with those of a random network (Sporns, 2011).

Criteria b and c were also best met at a threshold range from *r* = 0.20 to 0.27 (Fisher transformed).

### Modularity analyses

For all modularity analyses we used the Brain Connectivity Toolbox (http://www.brain-connectivity-toolbox.net/).

To decompose the RSN of Broca's area into its partitions, we used the Louvain algorithm (Blondel et al., 2008), since that algorithm allows to elucidate hierarchical modularity. That means that each partition or module detected at the highest analysis level can be divided into its sub-modules in a next analysis step and these sub-modules again into their sub-sub-modules and so on. The Louvain algorithm involves some heuristics, hence the outcomes, i.e., number of modules and the assignment of a node to a specific module or community, may vary from run to run. For that reason, we repeated the computation for 100 times and chose the most frequent outcomes.

The modularity Q quantifies the goodness by which a network can be optimally divided into non-overlapping modules, i.e., into clearly delineated subgroups of nodes which show more and denser association with each other than with the nodes of the other subgroups of the network (Newman, 2006; Rubinov and Sporns, 2011). In a first step, we explored the modularity of our empirical data as a positively and negatively weighted network without the necessity to define an arbitrary threshold. However, the contribution of positive and negative weights to the modularity of a network is not the same. Positive weights are of greater importance, because they assign a node explicitly to a module, whereas a negative weight implicitly excludes a node from a module (Rubinov and Sporns, 2011). The original mathematical definition for the goodness of a modularity partition Q by Newman (2006) does not take into account the different contributions of negative and positive weights. To assess the goodness of partition of the Louvain algorithm, we therefore chose for all modularity analyses the Q^*^ of Rubinov and Sporns (2011), which is able to deal with the unequal importance of positive and negative weights. Finally, to evaluate the significance of the Q^*^-values of the partitions of our empirical data, we used the algorithm of Rubinov and Sporns (2011) to model equivalent network null models and computed their Q^*^. Afterward, we tested the computed partitions and Q^*^-values for these null model networks against the empirical data using a *t*-test in combination of a bootstrapping procedure.

In addition, we also computed two measures that are able to describe the roles of the nodes in a module. The participation coefficient *P* characterizes the capacity of a node to influence the information flow between the other subgroups/modules of the network, while the within-module-z-score quantifies the influence of a node inside its module. Nodes, that influence the information flow between the modules in a significant way, are called connector hubs. Nodes influencing the information flow within the module in a significant way are termed provincial hubs (Rubinov and Sporns, 2010). Again, we computed both measures for each modularity analysis over the entire defined threshold range.

### Further graph analyses

All other graph analyses were performed using the graph toolbox that is implemented in conn 13l. The following graph analyses measures were investigated for our network over the entire threshold range from *r* = 0.20 to 0.27:

- Measures of segregation: The clustering coefficient *C* describes how close the relationship of the neighboring nodes of a node i are. The network's clustering coefficient, as well as an individual node's clustering coefficient can be interpreted as a measure of how resilient the network or the neighborhood of a node generally is when attacked (Rubinov and Sporns, 2010).
- Measures of integration: The characteristic path length describes the node i's capacity for information integration and is defined as the shortest sequence of edges from node i to node (Rubinov and Sporns, 2010).
- Measures of influence: The influence or centrality of a node can be measured by its degree K. Nodes with a high degree are densely connected with the other nodes of the network and may play an important role in ensuring that information flows efficiently between nodes (Rubinov and Sporns, 2010).

Betweenness centrality describes how many of the shortest path lengths pass through a node. This measure provides information about a node's centrality or influence in the network.

Nodes being very influential for processing information in the brain are called hubs. Generally, they are characterized by a high degree, as well as by high values of betweenness centrality (Rubinov and Sporns, 2010). For our analysis we defined nodes with a degree as well as a betweenness centrality with at least one standard deviation above the network mean as hubs. Nodes that qualified as hubs and also showed the highest participation coefficient of their respective module were defined as connector hubs.

## Results

### Results of the resting state analysis for broca's area (left pars triangularis and the left pars opercularis)

**Figure 1:**
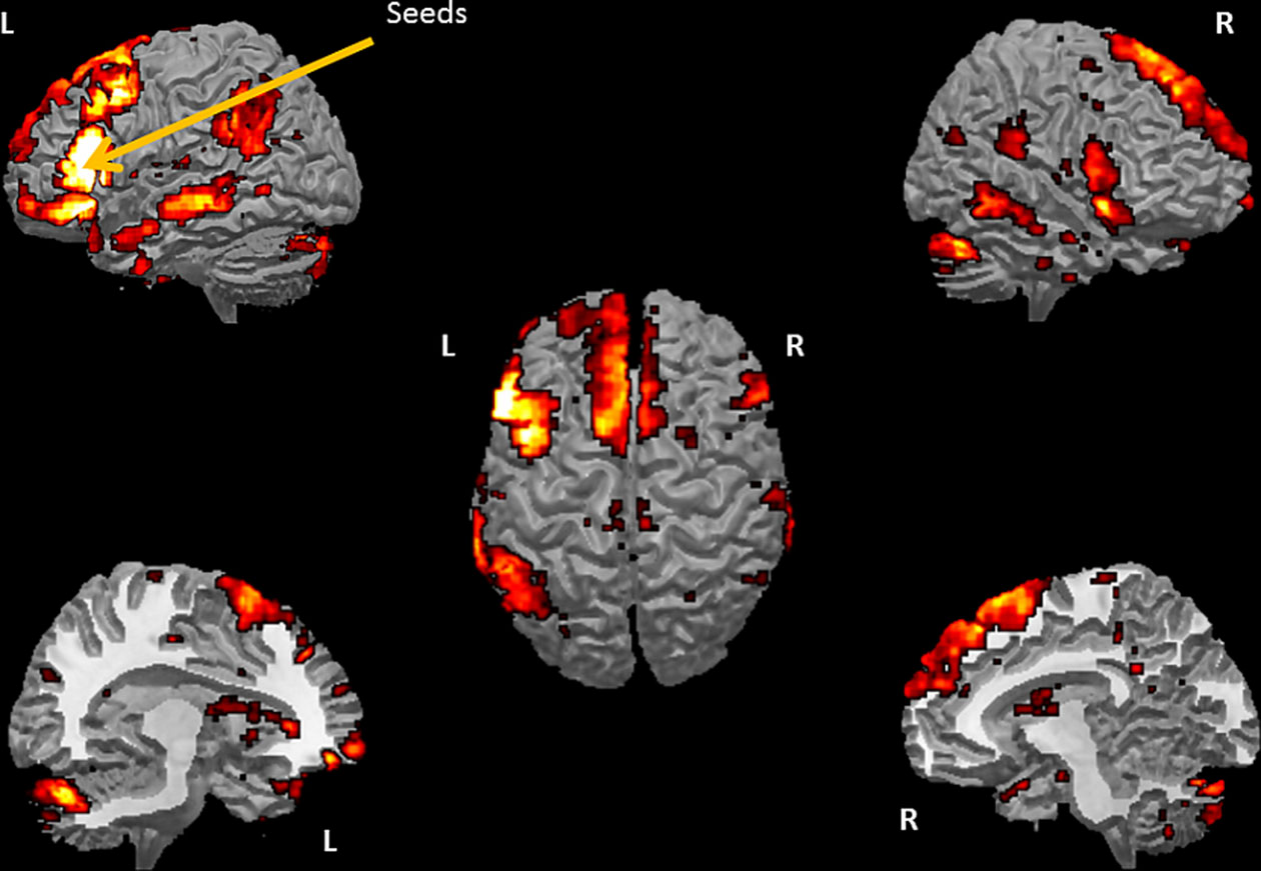
**Whole brain cluster map for the two seeds in Borca's area.** The figure presents the cluster map of the resting state analysis with seeds in the pars triangularis (−53 +20 +15) and the pars opercularis (−46 +16 +8) (Height threshold for peak voxels: *p* < 0.01 FDR and extension threshold for clusters: *p* < 0.01 FWE).

**Figure 2:**
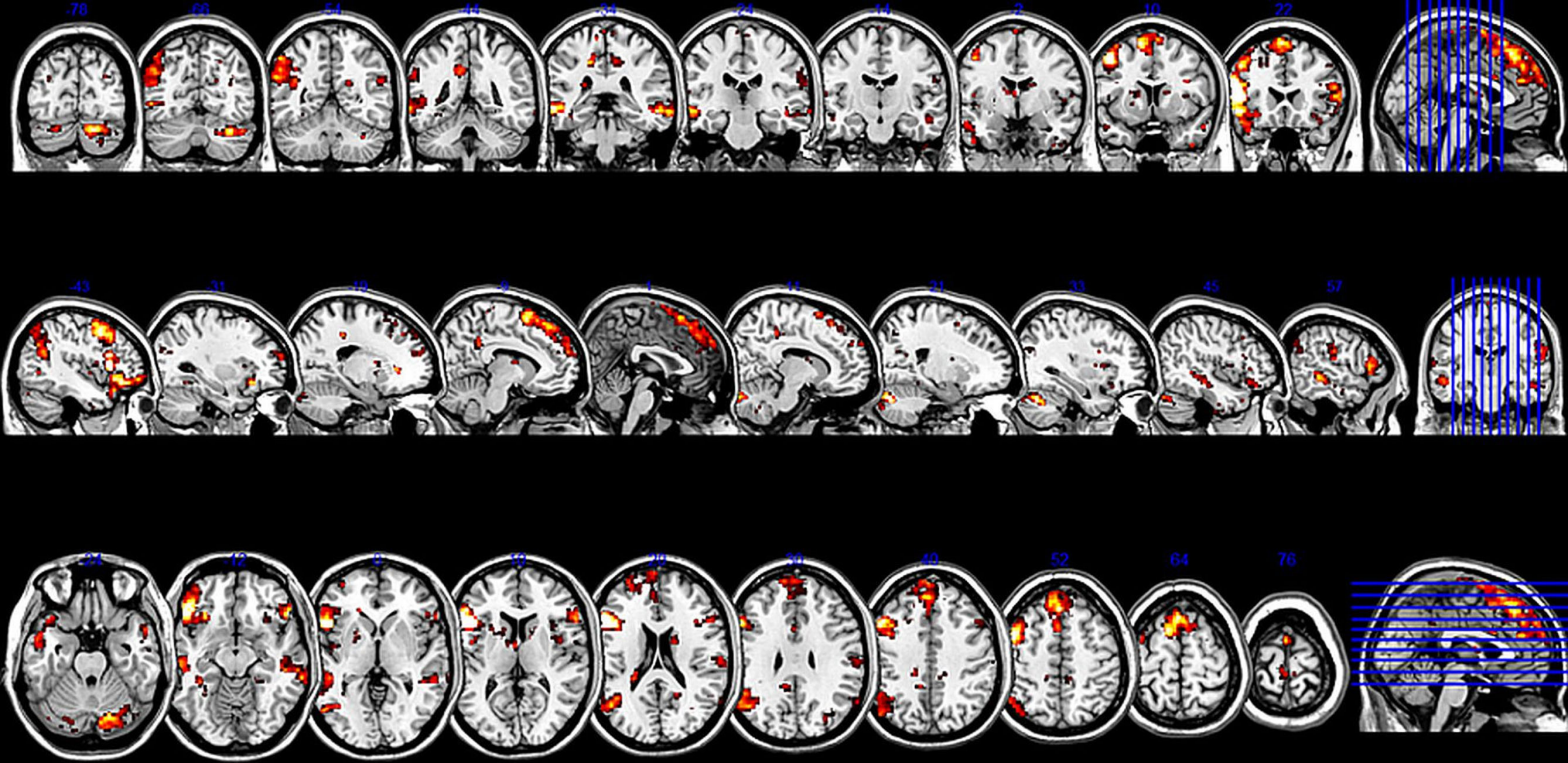
**Sliced version of the cluster map for the two seeds in Broca's area.** The figure shows coronal, sagittal, and axial slices of the cluster map of the resting state analysis with seeds in the pars triangularis (−53 +20 +15) and the pars opercularis (−46 +16 +8) (Height threshold for peak voxels: *p* < 0.01 FDR and extension threshold for clusters: *p* < 0.01 FWE).

The SCA cluster map (Figures 1 and 2) for the left pars triangularis (−53 +20 +15) and the left pars opercularis (−46 +16 +08) shows a total of 25 clusters (*k* = 56–6454 voxels). From these 25 clusters, 15 clusters are positively correlated with the two seeds (Table 1). The most extensive cluster (6454 voxels) is positively correlated and comprises the left premotor cortex, the left dorsolateral frontal cortex, the left dorsal frontal cortex, the left pars triangularis and opercularis, the left middle temporal gyrus extending into the left temporopolar area, the left inferior temporal gyrus, and the left superior temporal gyrus. The second most extensive cluster (4540 voxels) is also positively correlated and is bilateral, symmetrical, and covers the following areas: the bilateral medial superior frontal gyri, the left and the right dorsal frontal cortex, the dorsolateral frontal prefrontal cortex, and the bilateral premotor cortex. The third largest also positively correlated cluster (1988 voxels) contains the area from the left angular gyrus and left supramarginal gyrus to the left somatosensory association cortex. The fourth most extensive cluster (1394 voxels) is positively correlated and resides in the right cerebellum (lobule VIIa crus I). The fifth largest cluster (1103 voxels) is located in the right homolog of the two seeds. There are smaller positively correlated clusters in the right middle temporal gyrus (888 voxels) and the left posterior cerebellum (272 voxels).

**Table 1:**
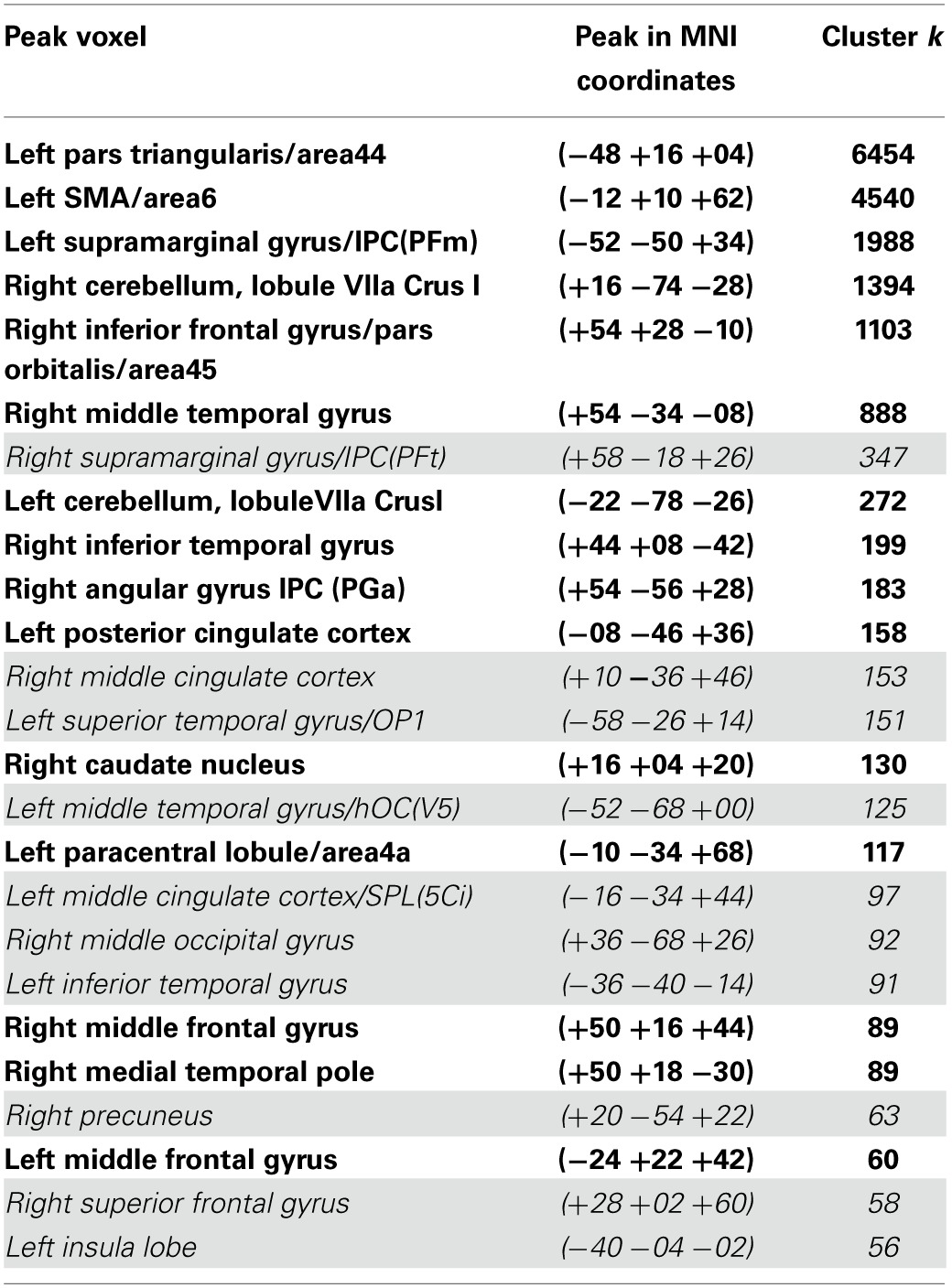
**List of all the resting state Broca's area network's clusters, which possessed seeds in the left pars opercularis (−46 1 16 18) and the left pars triangularis (−53 +20 +15) (peak height threshold: *p* < 0.01 FDR corrected; cluster extent threshold: *p* < 0.01 FWE corrected)**.

Bold characters mark the positively correlated clusters whose peak voxels were used to build the nodes for the subsequent graph analyses. Gray-shading and italic characters mark the negatively correlated clusters that were removed from all of the subsequent steps of analysis. The SPM Anatomy toolbox (Eickhoff et al., 2005) was used to identify the exact location of the peak-voxels.

Although their meaning is still under debate, there is evidence that anticorrelations in resting state data are not an artifact caused by the global signal regression step in the preprocessing used by certain pipelines as was suggested by Murphy et al. (2009), but have a real neurobiological basis (Fox et al., 2009; Chai et al., 2012; Whitfield-Gabrieli and Nieto-Castanon, 2012). The most extensive negatively correlated cluster covered the right supramarginal gyrus extending into the primary somatosensory cortex (347 voxels), regions of the right somatosensory association cortex and the dorsal posterior cingulate cortex (153 voxels), the left primary auditory cortex reaching into the supramarginal gyrus (151 voxels), the left fusiform gyrus (125 voxels), and a part of the left dorsal posterior cingulate gyrus and the somatosensory association cortex (97 voxels).

### Results of the ICA

As one of the aims of our study was to shed light on the role of the right hemisphere in language processing, it was essential, to confirm it was not an artifact, and especially to evaluate its relevance for language. For this purpose, we performed a purely data driven whole brain ICA with 70 components. The subsequent spatial sorting of the 70 independent components by a correlation analysis revealed that component 22 correlated significantly at *r* = 0.51 (*p* < 0.001, corrected for multiple comparisons) with the cluster mask from the previous SCA.

**Figure 3:**
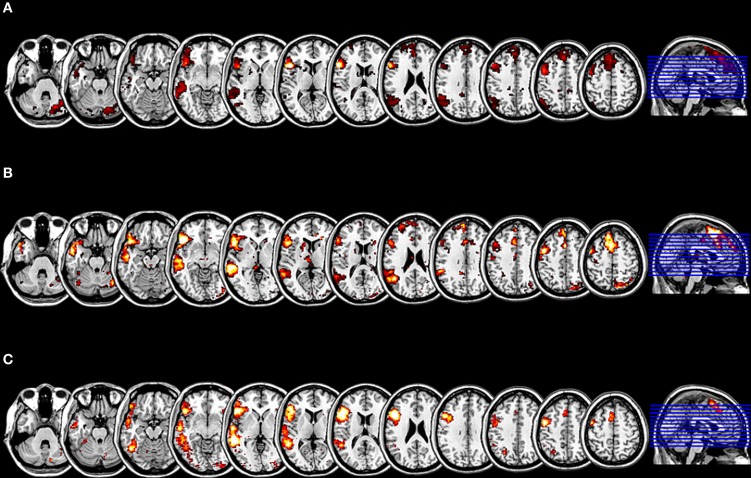
**Results of the evaluation of the functional plausibility of the language relevant RSN. (A)** The cluster map for the SCA with seeds within the pars triangularis and the pars opercularis, with an extension threshold for clusters of *p* < 0.01 FEW. **(B)** The t-mask for the ICA generated component 22 thresholded at *p* < 0.01 FDR. The SCA cluster map and the component 22 are significantly correlated (*r* = 0.51; *p* < 0.001 corrected for multiple comparisons). **(C)** The z mask for the feature “language” from the meta-analysis platform NeuroSynth thresholded at *p* < 0.001 FDR. The SCA cluster map and the z mask “language” are significantly correlated (*r* = 0.47; *p* < 0.001 corrected for multiple comparisons).

By means of the NeuroSynth framework, we computed for the two seeds which were used for the SCA a reverse inference z map as well as the posterior probability that the two seeds from the SCA are language relevant indeed. The seed located within the pars triangularis (−53 +20 +15) showed a z score of 8.17 corresponding to the likelihood that the feature “language” is used in a study given the presence of this activation, and posterior probability of 0.77 corresponding to the estimated probability of the feature “language” being used given the presence of an activation at this location. The seed located within the pars opercularis (−46 +16 +8) showed a z score of 4.69 for the reverse inference and a posterior probability of 0.69, that the feature “language” is used given an activation peak at this location. The result of the second spatial sorting of the 70 components with the corresponding z map for the feature “language,” that is based on an automated meta-analysis of 553 studies on language by the NeuroSynth platform, as the spatial template yielded a correlation with the component 22 of *r* = 0.47, which is highly significant at *p* < 0.001 (corrected for multiple comparisons). Thus, the highly significant correlations of independent component 22 with the cluster mask from the SCA as well as with the reverse inference z map for the feature “language” may indicate, that the here investigated network represents a language relevant function indeed (Figure 3).

### Results for the modularity analyses

#### Results of the modularity analysis—first level

**Figure 4:**
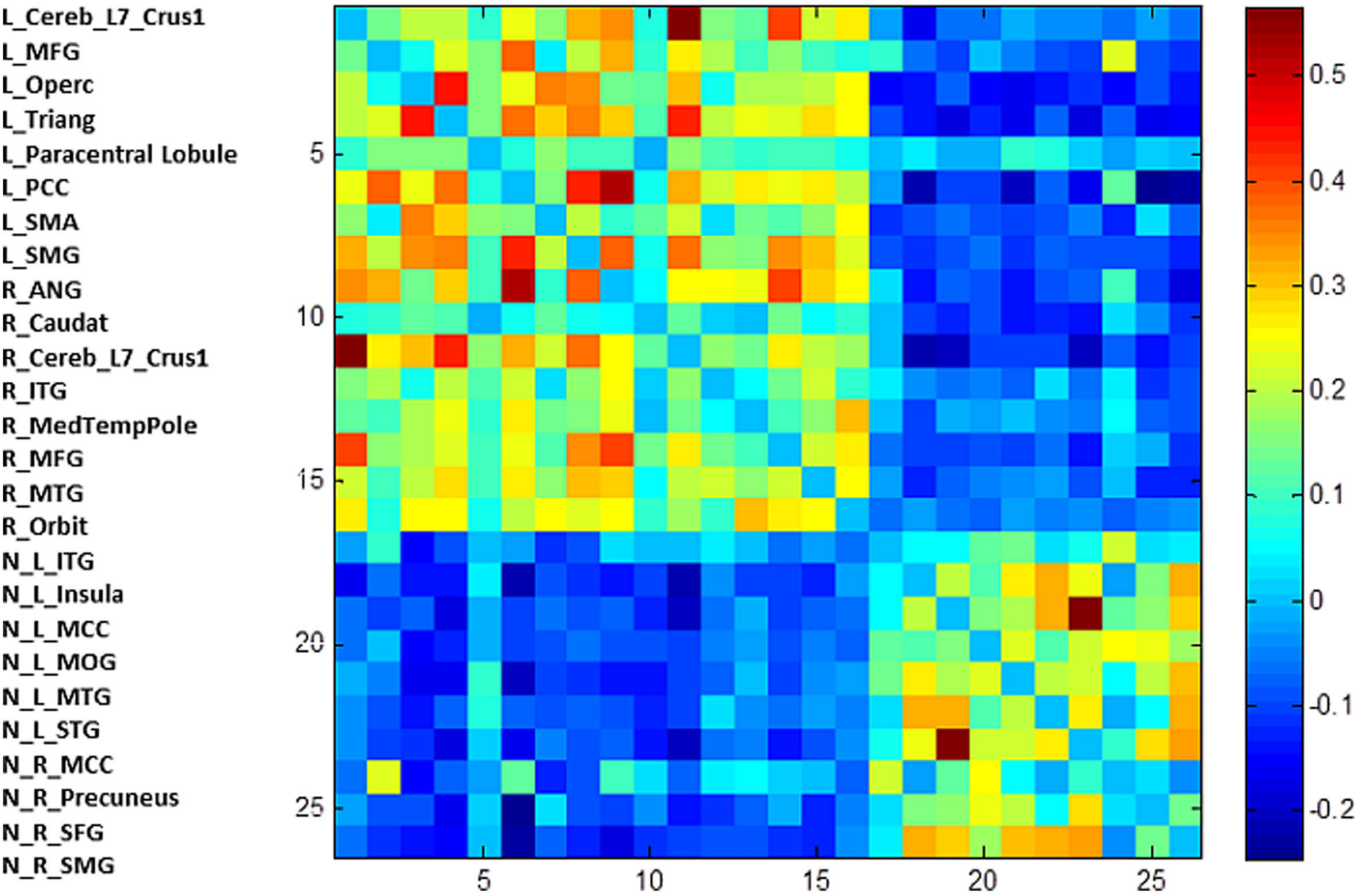
**The unthresholded adjacency matrix of the RSN.** The figure shows the adjacency matrix of the 25 positively and negatively correlated nodes. L, left; R, right; N, negative; Trinang, pars triangularis; MFG, middle frontal gyrus; PCC, posterior cingulate gyrus; SMA, supplementary motor area; SMG, supramarginal gyrus, Operc, pars opercularis; ANG, angular gyrus; Caudat, caudate nucleus; Orbit, pars orbitalis; ITG, inferior temporal gyrus; MedTempPole, medial temporal pole; MTG, middle temporal gyus; INS, insula; MCG, middle cingulate gyrus; STG, superior temporal gyrus; MOC, middle occipital cortex; Precun, precuneus; SFG, superior frontal gyrus.

**Figure 5:**
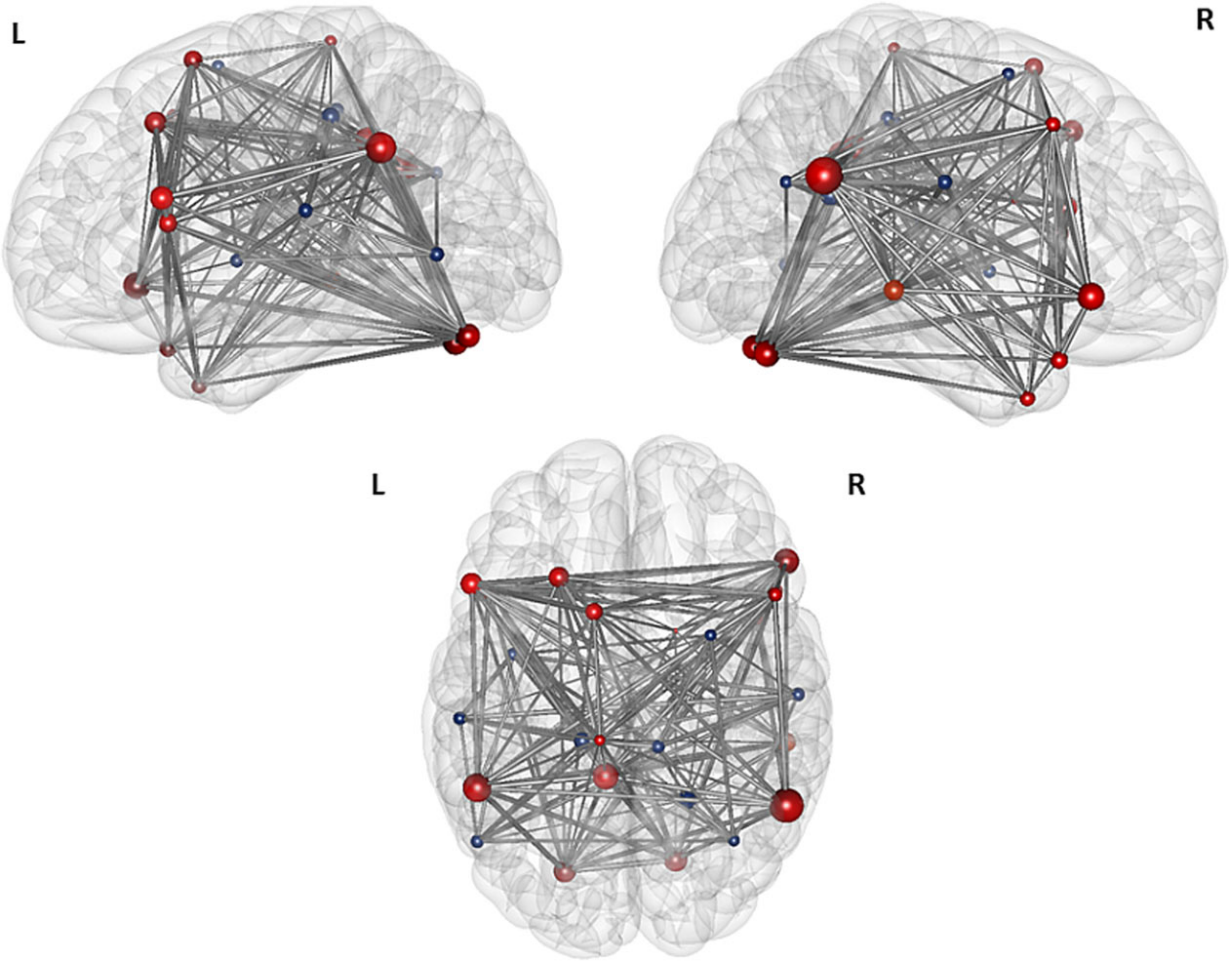
**Illustration of the modules after the first level modularity analysis.** The figure illustrates the result of the first partition of the 25 nodes network from top, left, and right orientation. The size of the nodes represents their global efficiency. The edges illustrate the *t*-values of all of the associations. The nodes that are positively correlated with the two original seeds are shaded red, the nodes that are negatively correlated with the original seeds are shaded blue.

The first modularity analysis of the unthresholded and positively and negatively weighted network showed two clearly distinct partitions: A first partition with all nodes that had shown positive correlation with the two original seeds of the SCA and a second partition with all nodes that had shown negatively correlated time series with the two original seeds (Figure 4). The unthresholded adjacency matrix (Figure 5) confirms that connectivity pattern: The nodes of the two partitions are positively connected among themselves, but there exist no positive connections between the two partitions with exception of the right precuneus which is positively correlated with the left middle frontal gyrus, the left angular gyrus, and the left posterior cingulate gyrus.

Based on the result of the first modularity analysis, and since there exists still no clear consensus about the meaning of anticorrelated nodes in a specific functional network, we decided to perform all further modularity and all graph analyses on the network of these 16 nodes, which had shown a positive correlation with the two seeds in the SCA.

#### Results of the modularity analyses—second level

**Figure 6:**
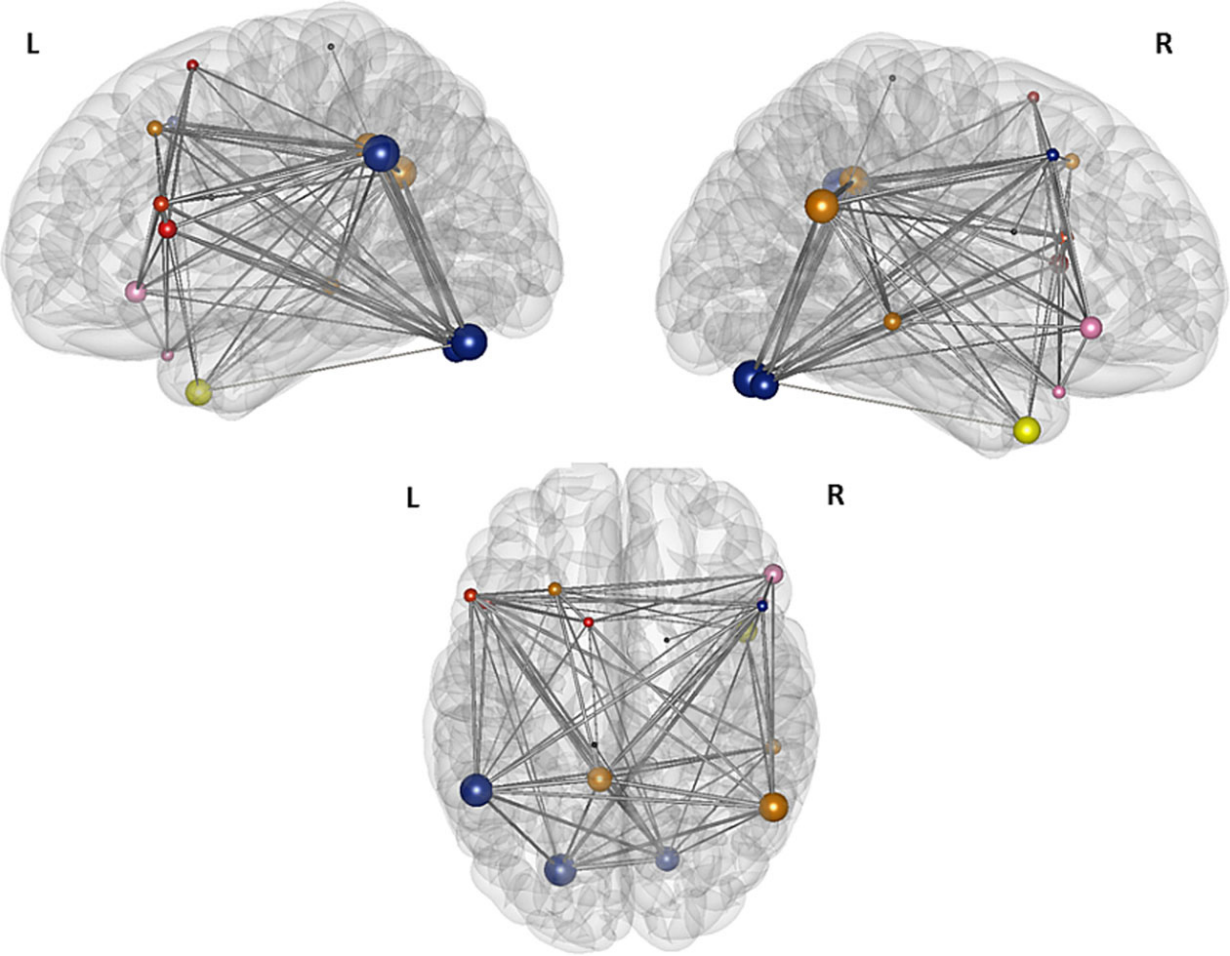
**Illustration of the modules after the second modularity analysis.** The figure illustrates the result of the partition of the 16 nodes network thesholded at (Fisher transformed) *r* = 0.27. The size of the nodes represents their global efficiency. The edges illustrate the *t*-values of all of the associations. The nodes of the left frontal module are shaded red, the nodes of the right frontal module are shaded rose—the left and the right frontal modules merge into one single module, the “language module” when the threshold gets more liberal; the nodes of the “connector module” are shaded blue and the nodes comprising the “DMN module” are shaded yellow. The nodes within the caudate nucleus, the right inferior temporal gyrus, and the left paracentral lobule are not assigned to any of the modules and are shaded black.

For the two sparsest thresholds (*r* = 0.27 and 0.26), we found four modules and three nodes, which were not assigned to any of the four partitions (Figure 6). The first module covers the frontal part of the brain and consists of three nodes in the left hemisphere, namely the nodes in the left pars triangularis, in the left pars opercularis, and in the SMA. The second, small, right sided module is also located in the frontal part of the brain and is comprised just of the two nodes: the right pars orbitalis and the right medial temporal pole. The third module is bilateral and made up of four nodes: The two nodes in the left and right cerebellum, the node in the right middle frontal gyrus, and the node in the left supramarginal gyrus. The nodes of the left posterior cingulate gyrus, the right angular gyrus, the left middle frontal gyrus, and the right middle temporal gyrus together belong to a forth bilateral module. The nodes located in the right inferior temporal gyrus, the left paracentral lobule, and the right caudate nucleus are not assigned to any of the four modules.

For the thresholds from *r* = 0.20 to 0.25, we found only three stable partitions. The first partition comprises the two former frontal modules and consists of the nodes in the left pars triangularis, the left pars opercularis, the left SMA, the right pars orbitalis, and the right medial temporal pole. A second module is almost identical with a previous module, but consists now of five nodes instead of four. These are the nodes in the left posterior cingulate gyrus, in the right angular gyrus, in the right middle temporal gyrus, in the left middle frontal gyrus, and newly the node in the left inferior temporal gyrus, which was not assigned to any of the modules before. The last module is identical with the former third module and comprises again the nodes in the left and right cerebellum, the node in the left supramarginal gyrus and the node in the right middle frontal gyrus. Once more, the nodes in the left paracentral lobule and the right caudate nucleus are not assigned to any of these three modules (Table 2).

**Table 2:**
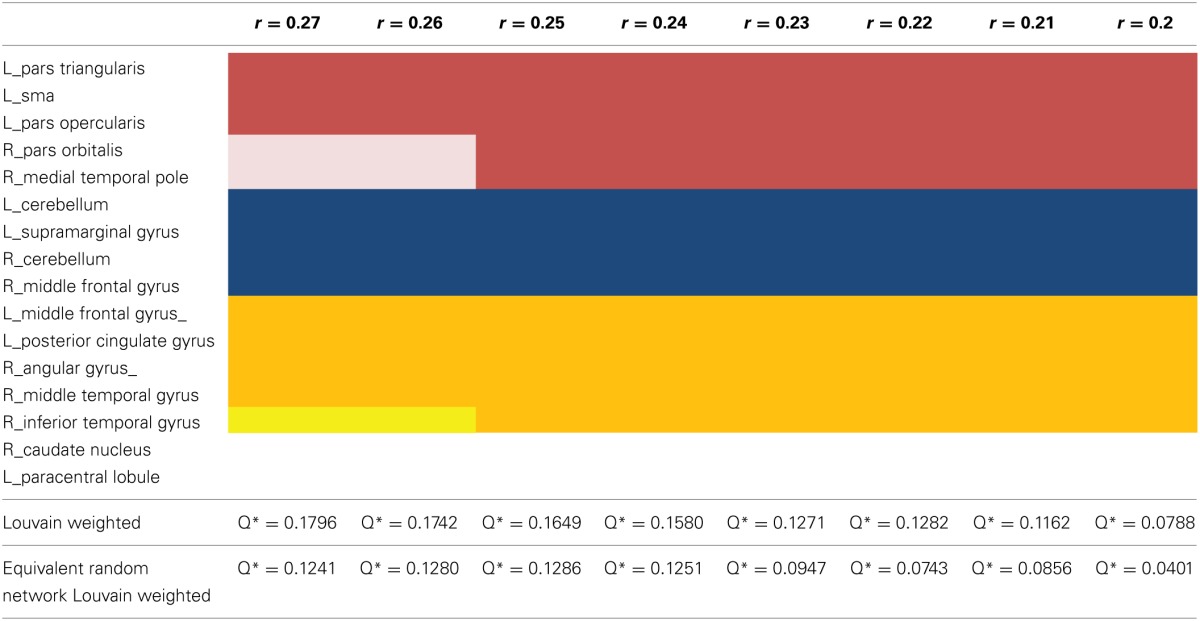
**Illustrating the result of the modularity analysis over the investigated threshold**.

The nodes of the “language module” are shaded red, respectively rose, the nodes of the “connector module” are shaded blue, and the nodes of the “DMN module” are shaded yellow. Additionally, the second lowest row shows the Q^*^-values for the partition of the empirical data, the lowest row the Q^*^ of an equivalent random network. The modularity algorithm used was the Louvain algorithm of Blondel et al. (2008).

**Figure 7:**
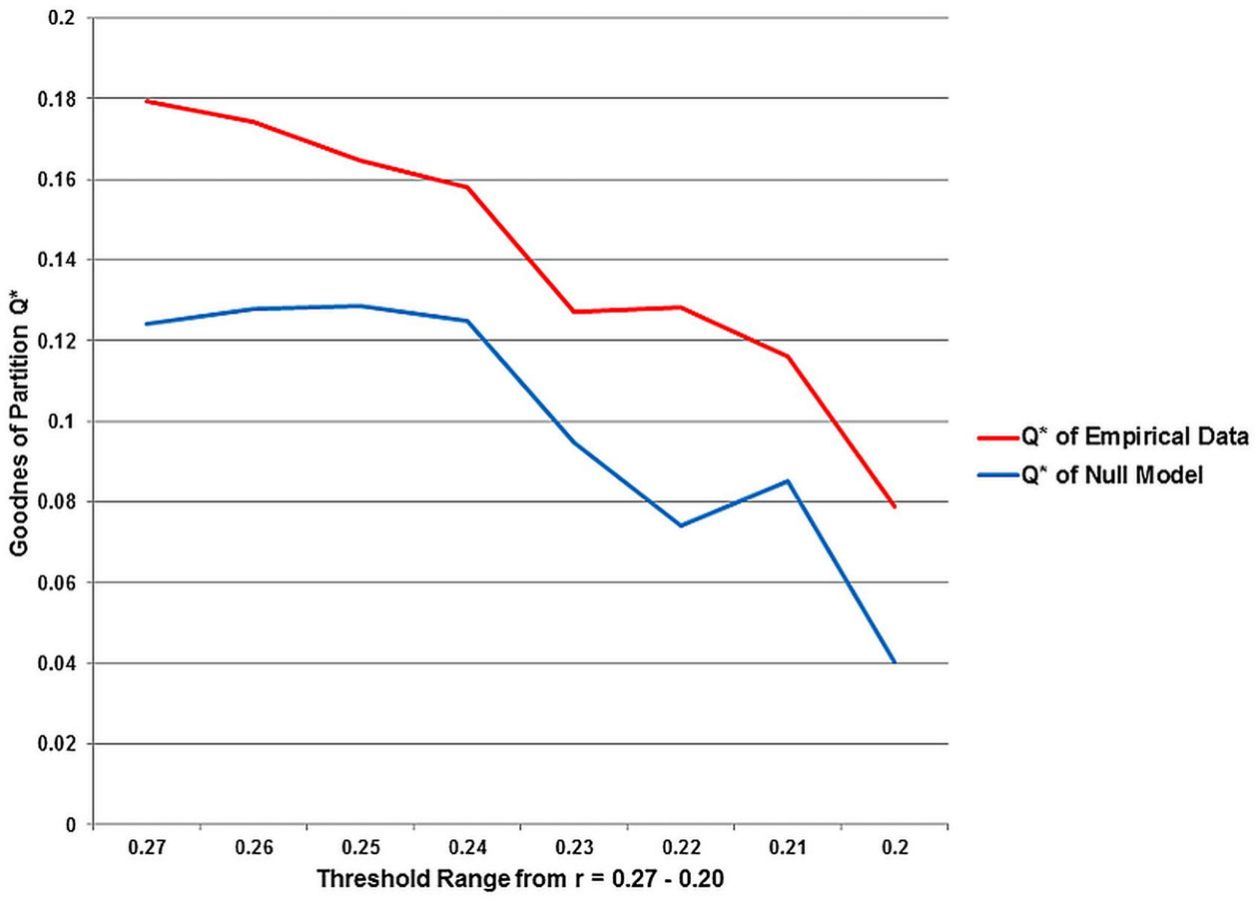
**The statistical significance of the three second level analysis modules.** The figure illustrates the values for the goodness of partition Q^*^ for the empirical data and for equivalent random networks over the entire investigated threshold. The value for Q^*^ shows the typical progress and decreases monotonically as the threshold gets more liberal. The Q^*^-values for the equivalent random networks are strikingly smaller than for the empirical data.

Q^*^, the measure for the goodness of a partition, and also the number of partitions decline as the chosen threshold gets more liberal, i.e., maximum modularity Q^*^ is observed for the sparsest network with the threshold at *r* = 0.27. The *t*-test showed, the modularity values for the equivalent null model networks are significantly lower (*p* = 0.028) over the entire threshold range (Figure 7).

We also computed the partition coefficient and the within z score for all 16 nodes over the entire threshold range from *r* = 0.27 to 0.20. The left posterior cingulate gyrus, the left pars triangularis and the right cerebellum in conjunction with the left supramarginal gyrus are the nodes with the highest values for the participation coefficient in their respective modules over the entire threshold range. There exists none so distinct pattern for the within z score. Although the role of the nodes for the within module interaction seems to change dynamically while lowering of the threshold, the left pars triangularis, the left posterior cingulate gyrus, and the left cerebellum have consistently high z-scores over the whole observed threshold range.

### Results of the further graph analyses

#### Global efficiency

The global efficiency of the overall network increases from 0.55 to 0.69 over the entire threshold range. The globally most efficient nodes are the left supramarginal gyrus and the left posterior cingulate gyrus; both manifest always a value that surpasses the overall network value by at least one standard deviation. Also, the nodes in the right angular gyrus, the left pars triangularis, the left pars opercularis, the right pars orbitalis, the left cerebellum, and the right cerebellum show values for global efficiency, that lie above the overall network value (Table 3A).

**Table 3:**
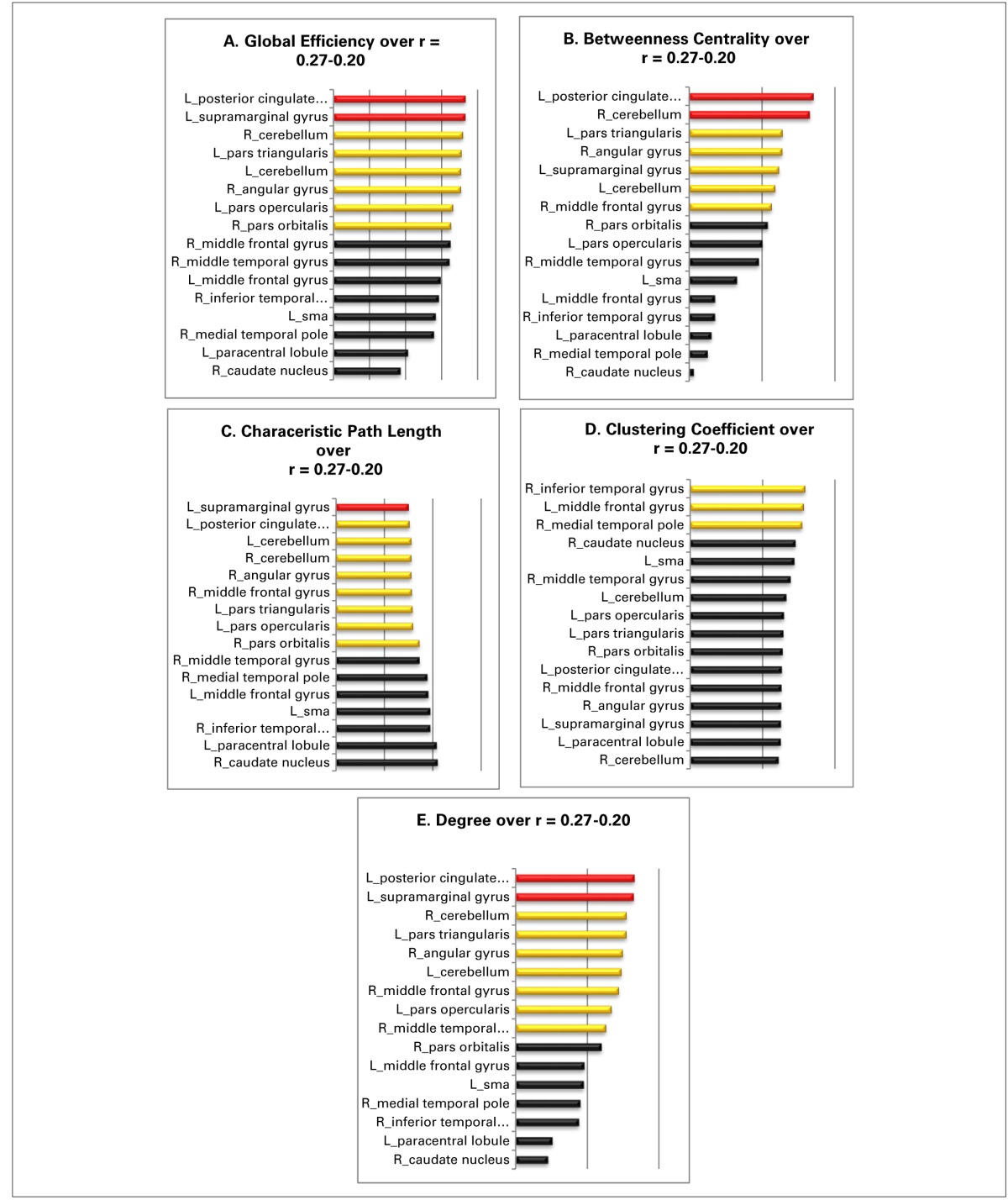
**The panels (A–E) illustrate the over the entire threshold averaged graph metrics of the resting state language network: red, nodes with a value at least one standard deviation over the network mean; yellow, nodes with a value higher as the overall network value for that specific graph metric; black, nodes with an equal or a smaller than the overall network**.

Only nodes with a significance level p < 0.05 are listed.

#### Betweenness centrality

The values for the betweenness centrality for the overall network decrease from 0.5 to 0.4 with more liberal thresholds. The nodes in the left posterior cingulate gyrus and the right cerebellum are again the only nodes that show values for betweenness centrality above one standard deviation over the entire investigated threshold range. The nodes in the left pars triangularis, the right angular gyrus, the left supramarginal gyrus, the right middle frontal gyrus, and the right cerebellum possess values for betweenness centrality, that lie all above the mean value for the overall network (Table 3B).

#### Characteristic path length

The values for the characteristic path length of the overall network cover a range from 1.82 to 1.62. The most central node of the network under investigation is the node in left supramarginal gyrus as its value for characteristic path length surpasses the network value at least by one standard deviation over the entire observed threshold range. The nodes in the left posterior cingulate gyrus, in the right angular gyrus, in the left pars triangularis, in the left pars opercularis, in the right pars orbitalis, and the nodes in the left as also the right cerebellum show all values above the network mean (Table 3C).

#### Clustering coefficient

The overall network values for the clustering coefficient increases from 0.64 to 0.7 as the threshold gets more liberal, i.e., as the threshold decreases from 0.27 to 0.20. The nodes in the right inferior temporal gyrus, the left middle frontal gyrus, and the right medial temporal pole are the only ones, which show constantly a value for the clustering coefficient above the mean of the overall network over the entire threshold range (Table 3D).

#### Degree

Over the threshold range, the overall network shows values from 5.01 to 6.97 for degree. The two nodes in the left posterior cingulate gyrus and the left supramarginal gyrus are the best connected nodes in the network as they both possess a degree which is ranked constantly one standard deviation above the network mean. The nodes in the right middle temporal gyrus, in the right angular gyrus, in the left pars triangularis as well as the node in the left pars opercularis, and the two nodes in the left and right cerebellum also show a value for degree over the entire threshold range (Table 3E).

Summary of the findings: Although the two original seeds, especially the node in the pars triangularis, show values for centrality and influence well above the network mean and may be characterized as important nodes within the network, there are two other nodes clearly qualifying as actual connector hubs in this network: The left posterior cingulate gyrus and the left supramarginal gyrus. Both nodes manifest not only the highest values for centrality and influence but their values were at least one standard deviation above the network mean and they therefore qualified as the two most influential nodes within the entire sub-network. Neither node is known to have a prominent role in language processing, hence we will discuss the interpretation of this finding in detail in the discussion.

## Discussion

Our aim was to better understand the role of the right hemisphere in language processing and to elucidate how the brain coordinates the interaction of the dominant left hemisphere with the right hemisphere. For this purpose, we performed a SCA from Broca's area represented by two seeds in the left pars triangularis and the left pars opercularis. The peaks of the resulting resting state map were then used to define nodes for subsequent GTAs in order to elucidate the relationships and dependencies of the involved regions. In addition, the relevance of the modeled RSN for language processing was assessed by means of a data driven ICA and the internet based meta-analysis platform NeuroSynth.

As predicted, the SCA cluster map for Broca's area revealed a spatial activity pattern that is very similar to the activation patterns found by previous task evoked fMRI studies on language. Also, it clearly illustrates the involvement of the right hemisphere. In line with our predictions, we found that 13 out of the altogether 24 clusters, respectively eight out of the 15 positively correlated clusters, were located in the right hemisphere. For the discussion of the GTA findings, we want to focus on the results of the second level modularity analysis.

### The “language module”—the role of the right broca homolog in language processing

On the second level, we conducted additional modularity analyses on the 16 nodes network over a threshold range from *r* = 0.20 to 0.27. The two sparsest networks (*r* = 0.27–0.26) could be partitioned into four modules: Two were frontal modules, one of them contained the left pars triangularis, the left pars opercularis, and the node within the left SMA. The other frontal module contained the right pars orbitalis, respectively the right Broca homolog, and the right medial temporal pole. With more liberal thresholds, these two modules merged into one single bilateral frontal module with totally five nodes which are all well-known for their involvement in language processing. Regarding our aim to elucidate the involvement of the right hemisphere in language the most interesting questions are the coordination of the information flow between the bilateral frontal module and the other modules and the role of the right pars orbitalis in this. In comparison to the two left sided Broca's area nodes, the right pars orbitalis showed always lower values for all graph parameters describing centrality and capacity for information integration. The node that had the highest participation coefficient in this module and hence coordinates the information flow with the other network partitions is the left pars triangularis. The pars triangularis showed also the highest within z score of all nodes in the module and thus directs as well the cooperation within the module which indicates that it is the most influential node in this frontal “language module” while all other nodes have supporting functions only. Nevertheless, the right-sided nodes possess a special position within this frontal “language module” in that they became more autonomous and formed a separate module as the thresholds got more rigorous. This finding is in agreement with an interpretation of Vigneau et al. (2011) that they suggested in their meta-analysis of the contribution made by the right hemisphere to language processing. Keeping in mind that our two seeds correspond to two activation peaks found in former fMRI studies of syntactical processing, it is interesting that Vigneau et al. (2011) found a cluster in the ventral part of the right IFG/pars triangularis that consisted of almost the same coordinates as the coordinates of our node in the pars orbitalis. According to Vigneau et al. (2011, p. 590), this right sided cluster is “specifically involved in syntactic tasks.” The authors reason that syntactical processing relies on interhemispheric cooperation between the two homolog frontal areas, but by itself the right inferior frontal gyrus is unable to decode syntactical information.

### The paradox of a “default mode module” in the language related RSN

The nodes in the left posterior cingulate cortex, the left middle frontal cortex, the right angular gyrus, and the right middle temporal gyrus constituted a third module that also incorporated the right inferior temporal gyrus as the thresholds got more liberal. At a first glance, the collection of these nodes in a language related network constitutes a bit of a conundrum. While the left middle frontal gyrus is consistently and reliably found to activate during word retrieval from semantics (Price, 2012), the posterior cingulate gyrus and the angular gyrus are both very prominent nodes of the default mode network (DMN) (Fox et al., 2005; Fox and Raichle, 2007) and one of the best established properties of the DMN is that it deactivates during active tasks like language processing. How can it be that we see nevertheless parts of this network in a language relevant RSN?

#### The function of the DMN

The function of the DMN, which is composed of regions along the anterior to the posterior midline, the lateral parietal cortex, the prefrontal cortex, and the medial temporal cortex (Buckner et al., 2009), is still unknown. Tasks that require mentalizing, internal planning of future behavior, autobiographical memory, theory of mind, or self-referential decision making (Buckner et al., 2008; Andrews-Hanna et al., 2010) are known to activate DMN nodes. All of these functions though have one characteristic in common: they all require an internal discourse that implies language processing. It is therefore possible that our participants were *actively* engaged in one of these above mentioned functions associated with the DMN. The seeds for our analysis were chosen especially because their coordinates were found to match the activation peaks of former fMRI studies examining syntactic processing. Thus, it is reasonable that only a part of the DMN—the part that is significantly correlated with our seeds—surfaces in our analysis. Consequently, the composition of this module may be a mixture of subcomponents pertinent to two or more RSNs, one of which is the DMN. Nevertheless, there are alternative explanations for that module.

#### The deactivation pattern of the DMN during tasks

Firstly, one of the best known features of the DMN is that it seems to be more actively engaged during task-free periods because it shows an increase of activation, while it deactivates and seems more passive whenever the brain is actively engaged in actual tasks. Li et al. (2012) investigated this activation pattern of the DMN by means of stochastic dynamic causal modeling analyses (DCM) on resting state data as well as on effective connectivity data. They could demonstrate that during an active cognitive task the DMN shows a general increase in extrinsic connectivity among its constituent regions that is simultaneously combined with a reduction of intrinsic self-inhibition within these very regions. As a matter of fact, this reduction of intrinsic self-inhibition means that the nodes of the DMN are more excitable (Li et al., 2012). Paradoxically, both phenomena together result in this deactivation, which was consistently observed in almost every task evoked fMRI study (Li et al., 2012). The increased effective connectivity of the DMN allows for faster switching from the idling mode to task mode as well as for an increase in sensitivity to exogenous inputs. The authors suggest that this specific dynamic of the DMN may rather promote the processing of sensory input and facilitate or monitor cognitive performance (Li et al., 2012). Based on this reasoning, a deactivation of the DMN during active language processing might indicate that the DMN is actively subserving this process and not disengaging from it. For these recent findings alone, i.e., the DMN is facilitating the performance of complex cognitive functions, we would also expect to see traces of frequent coactivation of the DMN with these other networks in resting state data.

But there is also evidence for an engagement of the DMN during active language processing. Seghier and Price (2012) investigated the functional heterogeneity of the DMN regions during several language tasks. They found that while the deactivation in the left angular gyrus was weaker during a semantic task than a perceptual matching task, the deactivation in the anterior part of the posterior cingulate gyrus, and the medial prefrontal cortex was stronger for the semantic task than for the perceptual matching task. If we interpret that finding in the context of the above mentioned results of Li et al. (2012) regarding the activation-deactivation pattern of the DMN, we have an indirect confirmation of our results which also clearly shows the importance of the posterior cingulate gyrus for language processing.

#### The dynamic interactions of the DMN during rest

There is also mounting evidence for quite dynamic interactions of the DMN during rest. Using MEG, De Pasquale et al. (2012) investigated the crossnetwork interactions of the DMN and other well-established RSNs during rest by analysing the epochs of transiently high within network band limited power (BLP) correlations. They were able to show that the DMN and the posterior cingulate gyrus in particular, exhibit the highest degree of transient β band BLP correlation with other networks - and one of them was a language related network.

Also, the results of quite a number of studies indicate that the DMN functions not as a monolithic entity, but is composed of various subcomponents, whose interactions and relationships change dynamically depending on the demands placed on them at the time (Andrews-Hanna et al., 2010; Spreng et al., 2010; Leech et al., 2011; Stawarczyk et al., 2011). Especially, the posterior cingulate gyrus that has been shown to be the most influential hub of the language processing network under investigation, seems to possess an exceptional position among the regions that constituent the DMN. Leech et al. (2012) elucidated the functional connectivity of the posterior cingulate gyrus using a novel extension of the multivariate ICA approach that is able to extract temporal distinct, but spatial overlapping signals in a data driven way from fMRI data. They were able to identify ten partial overlapping sub-regions, each sub-region having a distinct time course of the BOLD-signal. The time series of these sub-regions were correlated with the activity in other brain networks. Especially five sub-regions within the dorsal posterior cingulate gyrus showed intrinsic connectivity with brain regions that are normally not associated with the DMN and these functionally discrete networks included executive, attentional, motor, and language networks.

### The “connector module”—how the brain coordinates the interaction between the two hemispheres

The fourth partition was the most stable of all observed modules, it comprised the same four regions over the entire threshold range: the left supramarginal gyrus, the right middle frontal gyrus, and the left and the right cerebellum. The most prominent feature of this module is the fact that over the entire threshold range absolutely all four nodes possess values for influence and centrality that are among the highest. Beside the most influential node, the left posterior cingulate gyrus, the left supramarginal gyrus even qualifies as a second very important connector hub within the network under investigation. Both, the left supramarginal gyrus and the right cerebellum are the nodes with the highest participation coefficient in this module, that means these two nodes are supposed to coordinate the information flow between their own and the two other modules. When we look at the connectivity strengths, respectively the strength of the correlations, there emerges the following pattern: The left supramarginal gyrus demonstrates its highest correlation with the left posterior cingulate gyrus [*r* = 0.43 (Fisher transformed); *p* < 0.0001 FDR corrected] and the right cerebellum with the pars triangularis [*r* = 0.43 (Fisher transformed); *p* < 0.0001 FDR corrected]. Based on this findings we interpret the function of the third module as one of a “connector module” proper between the “language module” consisting of the nodes within the left pars triangularis, the left pars opercularis, the SMA, the right pars orbitalis, and the medial temporal pole and the “DMN module” comprising the posterior cingulate gyrus, the right angular gyrus, the right middle temporal gyrus, the left middle frontal gyrus, and the right inferior temporal gyrus.

### The language relevant RSN in relation with the networks of the whole brain

Hitherto, we discussed our findings strictly related to their meaning for language, now we want to look at them from a more global view. By performing an elaborated version of ICA, Doucet et al. (2011) parcellated the brain into 23 RSNs. A subsequent hierarchical cluster analysis revealed two anticorrelated large scaled systems at the highest level, one system (S1) could be associated with mainly internal processing or intrinsic activity, the other system (S2) with chiefly external processing or extrinsic activity and attention. Both systems could be further partitioned into smaller modules. S2 could be subdivided into two modules, one module consisting of regions known to subserve sensory, motoric and attentional functions, the other module including regions of the primary and secondary visual cortex. For our findings more relevant is the system S1 that could be partitioned into three modules. One sub-module (M1a) comprised regions constituting the DMN, the second sub-module (M1b) consisted of a group of fronto-parieto-temporal RSNs known to be involved in maintenance and manipulation of information. The third sub-module (M1c) included insular, frontal, supramarginal, and subcortical areas. Based on the observation that this sub-module was the only one from the S1 system that showed also a positive correlation with the M2a sub-module from the S2 system and thus could be said to occupy the position of a mediator between the two large scale systems the authors suggested that the function of this sub-module M1c may be best described as switching between the two large-scale systems and controlling the overall information flow (Doucet et al., 2011). Relating on the findings of Doucet et al., we would like to interpret the “language module” as a language-weighted subcomponent of M1b, the “DMN module” as a language-weighted subcomponent of M1a and our “connector module” as a language-weighted subcomponent of M1c.

## Limitations

At this point, we want explicitly to emphasize that all the results reported herein only apply to the language sub-network defined by the 16 nodes and the method used to define the nodes and does not apply to the whole brain. Likewise, when we describe the role of one of the nodes, e.g. as a hub, then this statement is only valid for this sub-network and does not refer to the role of that node in the network of the whole brain.

We investigated only resting state data, but there is no doubt that the networks of the brain are significantly modulated by an actual task. Therefore, an important next step will be to investigate fMRI data acquired during an actual language task as well as a new set of resting state data in order to replicate the here presented findings and to elucidate the modulations in the network when the brain is actively processing language. Since we reused for this study an already existing set of connectivity data that was restricted to male participants only, we will have the opportunity to include not only more participants but also to match the sample thus that both gender are represented equally.

## Conclusion

The brain does not operate reactively, but is constantly engaged in proactively and dynamically predicting future demands and to warm-up the network configurations needed to face these impending tasks. We aimed to investigate this functional baseline in order to better understand how the brain generates language and also the role of the right hemisphere in language processing. We performed a SCA of resting state data with two seeds in the left inferior gyrus, representing the partes opercularis and triangularis. The cluster map and the peak voxel of this SCA served as the foundation for a subsequent modeling of a graph network. By conducting further GTAs and a modularity analysis of this graph network, we could clearly demonstrate that the right hemisphere is relevant in language processing. We found three stable modules: A “language module” that was bilateral and composed of nodes representing clusters within known language areas, namely, the left perisylvian cortex, the left SMA, right homolog of Broca's area and the medial temporal pole. Of particular interest is the “DMN module” which consists of five nodes, two of them, the posterior cingulate gyrus and the angular gyrus, are very prominent DMN regions. We related the function of this module to recent findings that demonstrate that the role of DMN is probably to facilitate and to monitor the performance of complex cognitive functions as well as to promote the processing of external sensory input. The “connector module” is composed of nodes in the left supramarginal gyrus, the right middle frontal gyrus and the left and the right cerebellum. All of the “connector module's” nodes demonstrate high values in every graph theoretical measure that characterized the capacity for highly efficient parallel processing and for influencing information processing globally over the entire network. On account of these features the “connector module” is ideally suited to mediate between the “language module” and the “DMN module.”

## Author contributions

Angela M. Muller designed the study, assisted by the acquisition of the MRI-data, analyzed, interpreted the data, wrote the manuscript, gave final approval of the version to be published and is accountable for all aspects of the work in ensuring that questions related to the accuracy or integrity of any part of the work are appropriately investigated and resolved. Martin Meyer contributed to the design of the study and had also the lead in the data acquisition. Further, he supervised the analysis and the interpretation of the data. He revised the manuscript critically and gave final approval of the version to be published.

### Conflict of interest statement

The authors declare that the research was conducted in the absence of any commercial or financial relationships that could be construed as a potential conflict of interest.
